# Independent signaling by *Drosophila* insulin receptor for axon guidance and growth

**DOI:** 10.3389/fphys.2013.00385

**Published:** 2014-01-20

**Authors:** Caroline R. Li, Dongyu Guo, Leslie Pick

**Affiliations:** Department of Entomology and Program in Molecular and Cell Biology, University of MarylandCollege Park, MD, USA

**Keywords:** *Drosophila*, insulin receptor, Chico, Dock, RTK

## Abstract

The *Drosophila* insulin receptor (DInR) regulates a diverse array of biological processes including growth, axon guidance, and sugar homeostasis. Growth regulation by DInR is mediated by Chico, the *Drosophila* homolog of vertebrate insulin receptor substrate proteins IRS1–4. In contrast, DInR regulation of photoreceptor axon guidance in the developing visual system is mediated by the SH2-SH3 domain adaptor protein Dreadlocks (Dock). *In vitro* studies by others identified five NPXY motifs, one in the juxtamembrane region and four in the signaling C-terminal tail (C-tail), important for interaction with Chico. Here we used yeast two-hybrid assays to identify regions in the DInR C-tail that interact with Dock. These Dock binding sites were in separate portions of the C-tail from the previously identified Chico binding sites. To test whether these sites are required for growth or axon guidance in whole animals, a panel of DInR proteins, in which the putative Chico and Dock interaction sites had been mutated individually or in combination, were tested for their ability to rescue viability, growth and axon guidance defects of *dinr* mutant flies. Sites required for viability were identified. Unexpectedly, mutation of both putative Dock binding sites, either individually or in combination, did not lead to defects in photoreceptor axon guidance. Thus, either sites also required for viability are necessary for DInR function in axon guidance and/or there is redundancy built into the DInR/Dock interaction such that Dock is able to interact with multiple regions of DInR. We also found that simultaneous mutation of all five NPXY motifs implicated in Chico interaction drastically decreased growth in both male and female adult flies. These animals resembled *chico* mutants, supporting the notion that DInR interacts directly with Chico *in vivo* to control body size. Mutation of these five NPXY motifs did not affect photoreceptor axon guidance, segregating the roles of DInR in the processes of growth and axon guidance.

## Introduction

Receptor tyrosine kinases (RTKs) play diverse roles in development, differentiation, homeostasis and disease [reviewed in Lemmon and Schlessinger (2010)]. This diversity in biological function is exemplified by Insulin/Insulin-like signaling (IIS) pathways which have been implicated in a broad range of biological processes and diseases including diabetes, obesity, and cancer [reviewed in Nakae et al. (2001); Baserga et al. (2003); Kahn et al. (2005); Taguchi and White (2008)]. IIS impacts virtually all basic cellular processes, including transcription, translation, and cell growth, with specific effects on mitogenesis, glycogen synthesis, lipolysis, cell survival, and glucose uptake. How the different biological functions of single RTKs are executed—in different cell types, at different life cycle stages, or in response to different environmental cues—is not well understood.

Key components required to segregate the biological activities of RTKs are adapter proteins that link RTKs to discrete downstream pathways [reviewed in Kholodenko (2006); Murphy and Blenis (2006); Pawson (2007)]. Signaling is activated by ligand binding which induces a conformational change in the insulin receptor (IR), activating its kinase activity, resulting in autophosphorylation, substrate phosphorylation and the binding of adapter proteins to phosphorylated tyrosines [reviewed in Pessin and Frattali (1993); White (1998); Fisher and White (2004); Hou and Pessin (2007); Kohanski (2007); Taguchi and White (2008); Hubbard (2013)]. The insulin receptor substrates (IRS1–4) are key adapter proteins that mediate many IR downstream functions [reviewed in Taguchi and White (2008); Copps and White (2012)]. These substrates amplify receptor signals by interacting with a range of additional adapter proteins and enzymes. In addition to the IRS proteins, other receptor substrates have been identified including CBL, involved in glucose uptake (Hou and Pessin, 2007), SHC, involved in mitogenesis, and CEACAM1, involved in insulin internalization and degradation (Poy et al., 2002). Further complicating an understanding of the link between receptor activation and specific biological outcomes is the observation that different RTKs utilize many of these same adapter proteins to cause different biological “readouts” [reviewed in Lemmon and Schlessinger (2010)].

Here, we have made use of *Drosophila melanogaster* as a relatively simple but powerful genetic model system to investigate how a single RTK functions pleiotropically to cause distinct biological outcomes. In contrast to mammals, *Drosophila* harbor only one IIS-family receptor, the *Drosophila* insulin receptor (DInR) (Petruzzelli et al., 1986a,b) [reviewed in Garofalo (2002)]. DInR is similar in protein sequence to both human IR and IGF-1R (~26% and ~27% identity respectively and ~39% similarity) with highest similarity in the intracellular kinase domain, but was shown to autophosphorylate only in response to insulin (Fernandez-Almonacid and Rosen, 1987). Loss-of-function *dinr* mutations cause recessive lethality, demonstrating that DInR is essential for viability. Animals transheterozygous for combinations of *dinr* hypomorphic alleles are viable, but growth delayed and small (Fernandez et al., 1995; Chen et al., 1996; Bateman and McNeill, 2004; Colombani et al., 2005; Shingleton et al., 2005). DInR controls growth in a cell autonomous fashion: overexpression of DInR in the eye resulted in eye overgrowth due to increases in both cell size and cell number (Coelho and Leevers, 2000; Brogiolo et al., 2001; Leevers, 2001; Goberdhan and Wilson, 2003). These effects on growth are also seen for Chico, the *Drosophila* IRS1–4 homolog that acts downstream of DInR (Bohni et al., 1999). DInR and other IIS pathway members have been implicated in other biological processes including, but not limited to, regulation of lifespan and aging (Tatar et al., 2001; Hwangbo et al., 2004; Tatar, 2004; Mair et al., 2005; Piper et al., 2005, 2007; Piper and Partridge, 2007; Giannakou et al., 2008; Min et al., 2008; Partridge, 2008), locomotor activity (Belgacem and Martin, 2006), eating behavior (Wu et al., 2005; Lingo et al., 2007), oogenesis (Tatar et al., 2001; LaFever and Drummond-Barbosa, 2005; Hsu et al., 2008), heart function (Ocorr et al., 2007), nutrient sensing (Britton et al., 2002; Puig and Tjian, 2006), and metabolism, with surviving *dinr* transheterozygotes or heterozygotes displaying increased levels of whole body or circulating sugar (Shingleton et al., 2005; Belgacem and Martin, 2006).

DInR is highly expressed in the developing nervous system and we previously found that it is required for photoreceptor axon guidance (Song et al., 2003). In this previous study, we used a yeast two-hybrid screen to identify DInR intracellular partners. As bait for this screen, we used the intracellular portion of DInR, which autophosphorylated in yeast cells. This screen identified Dreadlocks (Dock) as a DInR partner. Dock had previously been shown to be required for photoreceptor axon guidance during development of the adult visual system in *Drosophila* (Garrity et al., 1996), suggesting that DInR might also play a role in this process. Our yeast two-hybrid assays showed that interaction with Dock requires DInR kinase activity and involves both the SH2 and the SH3 domains of Dock. This finding was consistent with *in vivo* rescue experiments showing that both SH2 and SH3 domains of Dock are required for photoreceptor axon guidance (Rao and Zipursky, 1998). Using the *eyFLP-FRT* system (Newsome et al., 2000) to generate homozygous *dinr* mutant tissue in a heterozygous background, we found that photoreceptor axon guidance was disrupted in these *dinr* mosaic animals. Whole animal *dinr* transheterozygotes showed similar, but more extreme defects, similar to defects seen in *dock* mutants. In contrast, animals carrying *chico* mitotic clones or whole animal *chico* mutants showed normal patterns of photoreceptor axon targeting. Since *chico* cells are small, similar to *dinr* clones, this result shows that the axon guidance defects seen for *dinr* mosaics are not a simple result of *dinr*-associated growth defects.

**Figure 1:**
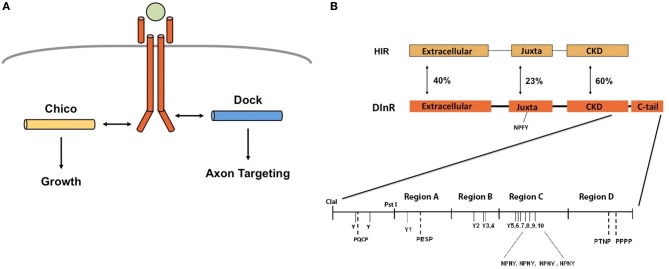
**DInR signals independently through Chico and Dock to control growth and axon guidance. (A)** We and others proposed that DInR, after DILP binding, signals independently through Chico to control growth and Dock to control axon targeting. Panel modified from Dickson (2003). **(B)** Schematic of DInR sequence with candidate binding regions indicated. The C-tail of DInR, previously shown to be required for interaction with Dock (Song et al., 2003), was divided into four regions (Regions A–D) for analysis. Dock is expected to interact with tyrosine residues (Y) via its SH2 domain and PXXP residues via its SH3 domains. Four NPNY sites in the C-tail and the juxtamembrane NPFY (NPXY-1351) were required for interaction with Chico in cell-based assays (Poltilove et al., 2000). All tyrosine residues in the C-tail are indicated in the figure.

On the basis of these results, we previously proposed (Song et al., 2003) that the roles of DInR in growth and axon guidance are independent and mediated by different adapter proteins: binding to Dock regulates axon guidance while binding to Chico controls growth (Figure 1A). DInR interaction with either Dock (Song et al., 2003) or Chico (Poltilove et al., 2000) *in vitro* requires the DInR C-terminal tail (C-tail), an extension absent in mammalian IR/IGF-1R (Fernandez et al., 1995; Ruan et al., 1995; Yenush et al., 1996). This C-tail contains multiple potential tyrosine phosphorylation sites and is required for DInR signaling in cell culture (Fernandez et al., 1995; Ruan et al., 1995; Yenush et al., 1996; Marin-Hincapie and Garofalo, 1999). The C-tail also contains YXXM motifs that mediate direct binding to PI3K in cell culture (Yenush et al., 1996), but rescue experiments in flies suggested that Chico is necessary to link DInR to PI3K for signaling and growth control *in vivo* (Oldham et al., 2002). Five NPXY motifs, one in the juxtamembrane region and four in the DInR C-tail, were shown to be important for interaction with Chico *in vitro* (Poltilove et al., 2000). Here, we used yeast two-hybrid assays to identify the regions of DInR that bind Dock. We found that the region of the DInR C-tail that binds Dock is distinct and separable from the region containing Chico interaction sites. We show that rescue of viability of *dinr* mutants requires kinase activity and one specific tyrosine residue in the C-tail. In contrast, a DInR protein carrying mutations in all Chico interaction sites rescued viability and axon guidance defects, but yielded growth defects similar to those seen in *chico* mutants. Finally, DInR proteins carrying mutations in identified Dock binding sites still rescued axon guidance defects, suggesting a high degree of redundancy for this function of DInR.

## Materials and methods

### Construction of *dinr* cDNA

A full-length *dinr* cDNA was assembled from genomic fragments as follows: a 7.4 kb genomic EcoRI-NheI fragment spanning the entire *dinr* coding region and including 770 bp of 5′ UTR, 9 introns and 191 bp of 3′ UTR was isolated from BAC48I01 (BACPAC, Oakland, CA). Subsequently, this EcoRI-NheI fragment was inserted into *pSP-luc^+^NF* (Promega, Madison, WI) to generate plasmid *pSPgdinr*. An EcoRI-KpnI fragment including exons 1–4 from *pSPgdinr* was inserted into *pSP72* (Promega, Madison, WI) generating plasmid *pSPgdinrEK* and introns 1, 2, and 3 were deleted sequentially by PCR (95°C for 1 min; 30 cycles of: 95°C for 30 s, 55°C for 30 s and 72°C for 6 min; 72°C for 10 min). PCR products were self-ligated after gel purification with GenElute agarose spin columns (Sigma, St. Louis, MO) to generate plasmid *pSPdinrEK*. Similarly, to remove introns 4–9, a KpnI-AflII fragment from *pSPgdinr* was inserted into *pSP72* to generate *pSPgdinrKAf*. Introns 4–7 were eliminated by replacing the KpnI-NsiI fragment of *pSPgdinrKAf* with a corresponding KpnI-NsiI cDNA fragment of *dinr* generated by RT-PCR using a SuperScript One-Step RT-PCR kit (Invitrogen, Carlsbad, CA). Total RNA for this reaction was isolated from 0 to 10 h *w^1118^* fly embryos using Trizol reagent (GibcoBRL, Carlsbad, CA). Introns 8 and 9 were deleted from *pSPgdinrKAf* by PCR, as described above. Together, these steps generated plasmid *pSPdinrMid*, which contains the KpnI-AflII coding region, lacking all introns. To generate a full-length *dinr* cDNA, the KpnI site outside the *dinr* gene in *pSPgdinr* was deleted by digestion with NheI and AatII, followed by a fill-in reaction with Klenow. Genomic EcoRI-KpnI and KpnI-AflII fragments were replaced by cDNA fragments from *pSPdinrEK* and *pSPdinrMid*, respectively, to generate *pSPdinr*, the final construct. Sequencing of *pSPdinr* confirmed that it matched the BDGP sequence FBgn0013984. We note that plasmids containing partial or full-length *dinr* cDNA were extremely unstable and could only be maintained in One Shot® TOP10 cells (Invitrogen, Carlsbad, CA).

### Construction of mutant *dinr* cDNAs

To test the function of regions of DInR in intracellular signaling and rescue of *dinr* mutant phenotypes, cDNAs were generated that were truncated or that carried specific point mutations in candidate adapter binding sites. To generate deletions, the AflII-StuI fragment from *pSPdinr* was replaced by PCR amplification of *pSPdinr* of the fragment of interest. Borders of the C-tail and deletions are shown schematically in Figure 1B. The start of the C-tail is at position Q1672. Deletions were generated to target large regions including tyrosine and PXXP residues that are candidate protein interaction motifs in the C-tail. Deletions were generated by standard PCR reactions and removed the following regions: ΔAB removed amino acids D1611 to K1825; ΔCD removed S1860 to P2131; ΔD removed D2060 to P2131; ΔA removed D1611 to L1742; ΔBCD removed T1760 to P2131; ΔABC removed D1611 to S2042. Note that ΔA constructs removed a portion of the kinase domain, beginning at D1611, in addition to the A region. Also, for constructs missing the D region, the C-terminal end of DInR, from amino acids R2132 to A2144, is present upstream of the MYC3 tag. This region includes a PPPP sequence (P2133-P2136). The following point mutations were generated: DInR-KA (K1405→A) in the kinase domain; Y1F (Y1714→F) in the C-tail; Y2F (Y1776→F) in the C-tail; Y3,4F (Y1790→F, Y1793→F) in the C-tail; LESL (P1724→L, P1727→L) in the C-tail; Y7F (Y1965→F), Y8F (Y1989→F), Y9F (Y2005→F), Y10F (Y2026→F) and combinations thereof, in NPXY sites in the C-tail. To generate these point mutations, a PCR product of the *dinr* C-tail from plasmid *DInRCKD-PAS2-1* (Song et al., 2003) was subcloned into the vector *pSP72* at its ClaI/KpnI site to yield *pSPdinrMT*. Site-directed mutagenesis of *pSPdinrMT* was done by PCR (95°C for 2 min; 18 cycles of: 95°C for 30 s, 55°C for 1 min and 68°C for 8 min). The PCR product was digested with DpnI for 1 h at 37°C to destroy any unmutagenized template plasmid present, and was transformed into XL-1 competent cells. All mutations were confirmed by DNA sequencing. The ClaI-KpnI fragments with various *dinr* mutations were shuttled from *pSPdinrMT* into *pAS2-1OFCT* for yeast two-hybrid assays. Primer sequences available upon request.

To generate transgenic *Drosophila*, full-length, partial or point mutation-containing *dinr* cDNAs were inserted into *pUAST*-*dinrMYC3*, a P-element vector which includes a 102 bp region encoding a 3X Myc tag to generate in-frame C-terminal fusions. This vector was generated as follows. The AflII-NheI fragment in *pSPdinr* was replaced by the PCR fragment of *pSPdinr* amplified using two primers, P51 and Srf2, and digested with AflII/NheI to introduce an SrfI site so that the MYC3 tag could be inserted into the vector. The newly generated plasmid was named *pSPdinrM*. A MYC3 tag was excised from the plasmid *pSRL-hSNT MYC3* (a gift from Dr. Mitch Goldfarb, Hunter College) and subcloned into the SrfI/NotI site of *pSPdinrM*, generating *pSPdinrMYC3*. The *dinrMYC3* cDNA from *pSPdinrMYC3* was subcloned into the EcoRI/NotI site of *pUAST* to generate the full-length *pUASTdinrMYC3* plasmid. *dinr* cDNAs carrying deletions (ΔABC, ΔAB, ΔCD) were inserted into *pUASTdinrMYC3* by replacing the BsiWI-NotI fragment of *pUASTdinrMYC3*. Point mutations in the C-tail of *dinr*, generated in *pSPdinrMT*, were moved into *pUASTdinrMYC3* by the replacement of the AflII-NotI fragment.

To test the one NPFY motif in the juxtamembrane region, *pUASTdinr(JM-NPFF)MYC3* was generated, in which the tyrosine in the juxtamembrane NPFY motif was changed to a phenylalanine (Y1354→F). Site-directed mutagenesis to change the TAT codon for tyrosine to the TTT codon for phenylalanine was carried out with standard methods using *pSPdinrMYC3* and Vent polymerase (NEB, Ipswich, MA). Then, a ~7 kb fragment spanning the entire *dinrMYC3* coding region, and thus containing the mutated juxtamembrane NPFF site, was released from the mutated *pSPdinrMYC3* plasmid with NotI and EcoRI; this fragment was inserted into the NotI and EcoRI sites of *pUAST*-*dinr(Y7,8,9,10F)MYC3*, replacing the entire *dinr(Y7,8,9,10F)MYC3* coding region. *pUASTdinr(5NPXF)MYC3* was then made by excising a ~4 kb fragment containing the 4 mutated NPXF sites in the C-tail from *pUASTdinr(Y7,8,9,10F)MYC3* using AflII and inserting it into the AflII site of *pUASTdinr(JM-NPFF)MYC3* to replace the AflII fragment. The orientation and sequence of each *dinr* variant was verified by sequencing.

### Yeast two-hybrid assays

Yeast two-hybrid assays were performed as described (Song et al., 2003) using *pAS2-1OFCT* carrying either wild type or mutant versions of DInR and *pACT-Dock* (Song et al., 2003). One hundred microliters of saturated culture was inoculated into 3 ml of fresh media and grown to mid-log phase. One milliliter of culture was spun down. The pellet was suspended completely in 200 μ l of 10 mM Tris (pH 7.5)/0.05% Triton X-100. The samples were stored at −80°C. Frozen samples were allowed to thaw slowly on ice before analysis. One milliliter of ONPG solution was added and mixed by inverting several times. The reaction was carried out at 30°C. When the color changed to medium-dark yellow, the reaction was stopped by adding 500 μ l 1 M Na_2_CO_3_ and OD_420_ was measured. The β-galactosidase activity was calculated with the formula: β-Gal units = [OD_420_(absorbance by reaction product) × 1000]/[OD_600_(sample cell density) × (1 ml) × *t*(time of reaction)]. Assays were repeated at least three independent times using at least 3 samples for each point in each assay.

### Genetics and phenotypic analysis

Transgenic flies were generated by Rainbow Transgenic Flies, Inc. (Camarillo, CA) by standard P-element mediated transformation. Multiple independent transgenic lines were generated for each construct whenever possible. Transgenic lines carrying insertions on chromosome II were used for rescue experiments. Transgenic lines for rescue experiments expressed DInR proteins at similar levels, determined using a modification of the method of Ronshaugen et al. (2002).

Genetic crossing schemes used to generate stocks for the *dinr* rescue experiments are available upon request. For the following experimental crosses, parental flies were removed as necessary to prevent overcrowding of the progeny to be used for analysis. For the lethality rescue analysis, *arm-GAL4/arm-GAL4;FRT82Bdinr^273^/TM3Sb,armGFP* virgin females were crossed to *UAS-X/(UAS-X* or *CyO);dinr^ex15^/TM3Sb,armGFP* males. Adult progeny that had eclosed were scored for their bristle phenotype: either *Sb* or non-*Sb*. In the case that the *UAS-X* construct to be tested was homozygous lethal and had to be used in crosses with a *CyO* balancer, only non-*CyO* eclosed adult progeny were scored for their bristle phenotype.

For the growth defect rescue analysis, *arm-GAL4/arm-GAL4;FRT82Bdinr^273^/TM3Sb,armGFP* virgin females were crossed to *UAS-X/(UAS-X* or *CyO);dinr^ex15^/TM3Sb,armGFP* males. Eclosed non-Sb, non-CyO adult male or female progeny were collected separately in fresh food vials and were individually weighed in an ATI Cahn C-33 microbalance approximately 3–18 days after eclosion.

For the photoreceptor axon guidance rescue analysis, *arm-GAL4/arm-GAL4;FRT82Bdinr^273^/TM6BTb,GFP* virgin females were crossed to *UAS-X/UAS-X; dinr^ex15^/TM6BTb,GFP* males. Non-Tubby progeny at the wandering third instar larval or white prepupal stages were analyzed.

For SEM studies, adult flies were decapitated. Heads were fixed in 2.5% glutaraldehyde overnight at 4°C, washed 3 × 30 min. with 0.1 M PBS, dehydrated in ascending acetone grades and then critical point dried. They were then mounted on studs in the desired orientation under a stereo binocular microscope and coated with gold (thickness 30–35 nm). Scanning was done on SEM mode in an AMRAY 1820D electron microscope at 15 kV.

### Photoreceptor axon guidance analysis

Eye-brain complexes were dissected from third instar larvae or white prepupae in phosphate-buffered saline (PBS). A standard protocol kindly provided by C. H. Lee was generally followed for the staining of eye-brain complexes with *m*onoclonal *a*nti*b*ody 24B10 (MAb24B10): eye-brain complexes were fixed in 2% paraformaldehyde in a lysine-phosphate buffer containing 0.25% sodium m-periodate, washed in 0.5% Triton-X-100 in PBS (PBT), blocked in 10% normal goat serum (NGS) in PBT, incubated in 1:200 MAb24B10 in 10% NGS in PBT at 4°C overnight or longer, washed in PBT, incubated in 1:200 HRP-conjugated goat anti-mouse antibody in 10% NGS in PBT at room temperature for at least 2 h, washed in PBT, incubated in DAB, washed in PBS, and cleared and mounted in 70% glycerol in PBS. MAb24B10 specifically stains the cell bodies and axonal membranes of differentiated photoreceptors in *Drosophila melanogaster* and was originally generated by Zipursky et al. (1984). MAb24B10 used in our experiments was purchased from the Developmental Studies Hybridoma Bank at The University of Iowa.

## Results

### The DInR C-tail harbors separate binding sites for dock and chico

As described above, we proposed that DInR signals independently through Dock and Chico to regulate axon guidance and growth, respectively (Figure 1A). To test this, yeast two-hybrid assays (Y2H) were used to identify potential Dock interaction sites in DInR. Because Dock interaction with DInR required the C-tail (Song et al., 2003), a series of small deletions and point mutations in DInR was generated in this portion of DInR (Figure 1B; Materials and Methods). For the deletion series, the C-terminal portion of DInR was divided arbitrarily into 4 regions (Regions A-D, Figure 1B) which were fused to the rest of the intracellular domain of DInR to allow for autophosphorylation in yeast (see Song et al., 2003). Region A includes a portion of the highly conserved kinase domain (between the ClaI and PstI sites indicated in Figure 1B), as well as the N-terminal portion of the C-tail that harbors two potential Dock interaction sites, Y1714 and a PESP motif at position 1724. Region B harbors three tyrosines (Y1776,1790,1793) that could potentially interact with Dock. Region C includes 6 tyrosines, the last four of which are embedded within NPXY consensus sequences previously shown to be involved in Chico interaction (Poltilove et al., 2000). Finally, region D contains one PXXP sequence, potentially able to bind Dock's SH3 domains. These tyrosine residues, all indicated in Figure 1B, are the only tyrosine residues present in the DInR C-tail.

**Figure 2:**
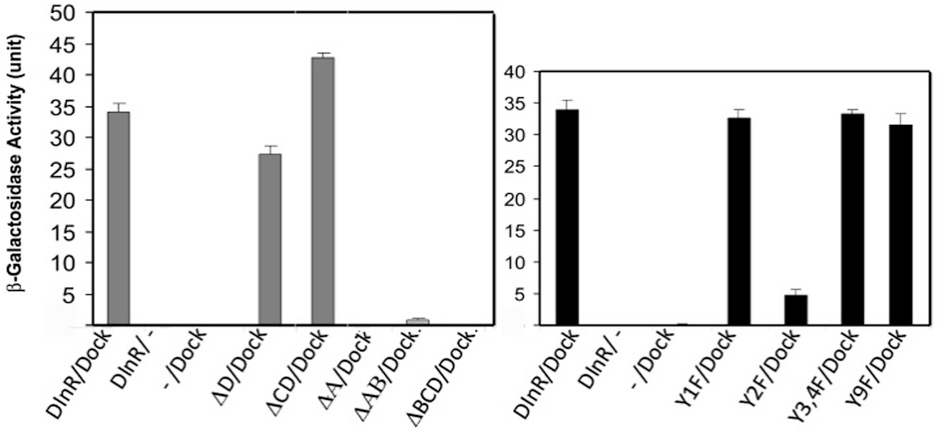
**Yeast two-hybrid assays identify candidate Dock interaction sites in the DInR C-tail.** pACT-Dock was tested for interaction with pAS2.1 containing the DInR intracellular domain or variants thereof, as indicated. β-galactosidase activity is shown. Negative controls: pAS2.1-DInR with empty pACT (−); empty pAS2.1 (−) with pACT-Dock. Error bars indicate standard deviation.

As shown in Figure 2, the full-length DInR intracellular domain interacted strongly with Dock. DInR-ΔD, which lacks the D region, and DInR-ΔCD, which lacks both C and D regions, interacted as strongly with Dock as full-length DInR. This result suggests that regions A and B are sufficient for the DInR/Dock interaction. Consistent with this, proteins lacking the A (DInR-ΔA) or A and B (DInR-ΔAB) regions did not interact detectably with Dock. However, the A region alone was not sufficient for interaction, as DInR-ΔBCD did not interact detectably with Dock. Note that the deletion of the A (DInR-ΔA) region alone suggests that the B region is also not sufficient for Dock interaction; however, as conserved regions of the kinase domain were removed in DInR-ΔA, we cannot make a firm conclusion about the C-tail requirements in this case.

To further investigate the sequence motifs necessary for DInR/Dock interaction, point mutations were generated in tyrosine residues in candidate adapter protein binding sites in the C-tail. As shown in Figure 2, mutation of Y1714 to F (DInR-Y1F) in region A did not significantly decrease interaction with Dock. In contrast, mutations of Y1776 in region B (DInR-Y2F) greatly decreased Dock binding. Mutations of the other tyrosine residues in the B region (double mutation of Y1790 and Y1793; DInR-Y3,4F) did not alter Dock interaction. Finally, point mutations of the tyrosine residues in the C region had modest or no effect on Dock interaction, shown here for Y2005F (DInR-Y9F). Together, these results suggested that DInR interaction with Dock requires the B region, in particular, Y1776 in this portion of DInR. Since the A region was also required for Dock interaction, but the tyrosine in this region was not, and since Dock function *in vivo* involves both SH2 and SH3 domains (Rao and Zipursky, 1998), these results suggested that the PXXP motif in the A region also interacts with Dock.

### Full-length Myc-tagged DInR promotes growth and rescues viability

**Figure 3:**
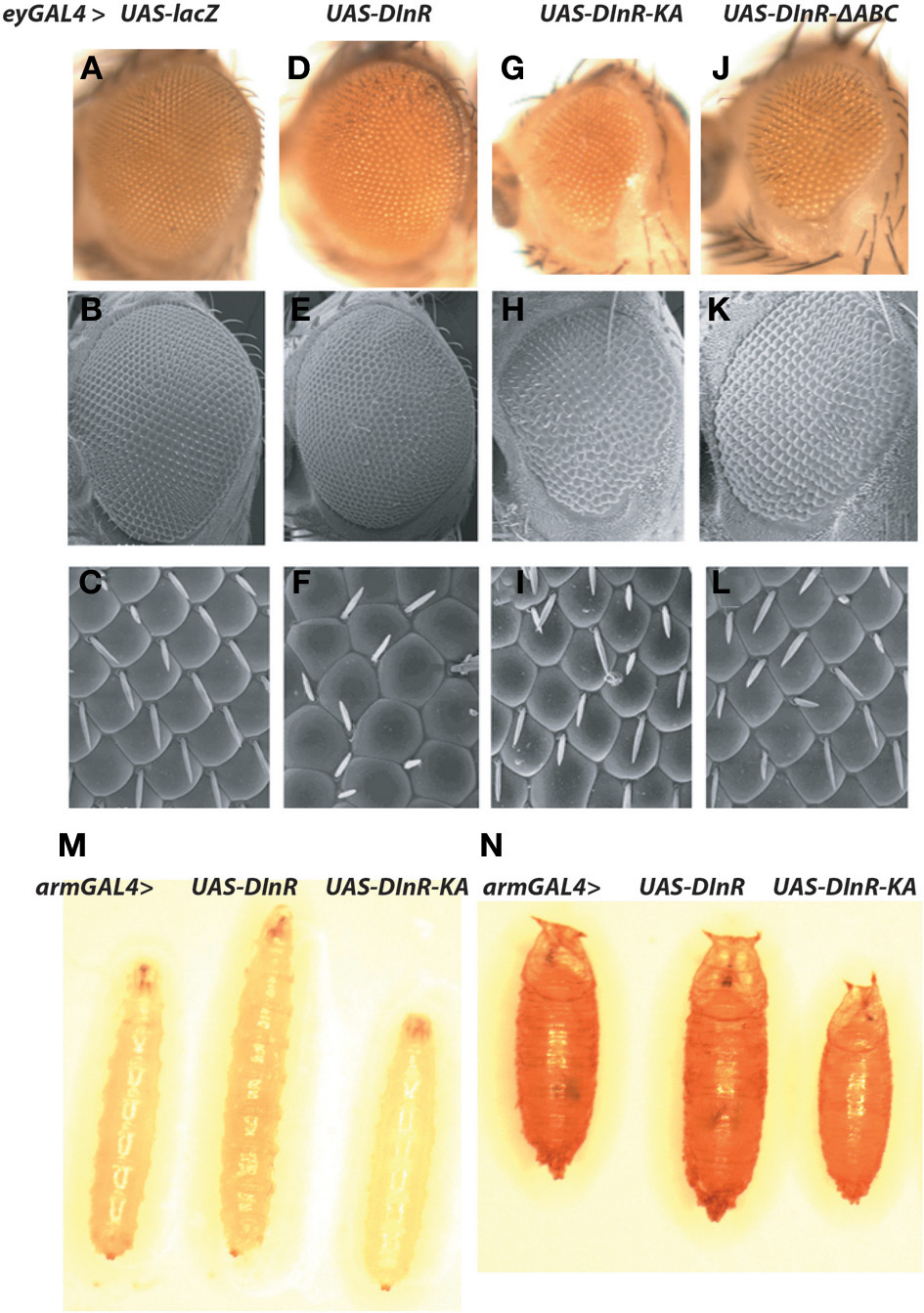
**Effects of ectopic expression of DInR on tissue-specific and whole animal growth.** Effects of ectopic expression of full-length Myc-tagged DInR or variants was tested using the *UAS/GAL4* system. **(A–L)** Expression driven by an *eyeless-GAL4* driver. **(A–C)** Control, *UAS-lacZ*; **(D–F)** *UAS-DInR*; **(G–I)** *UAS-DInR-KA*; **(J–L)** *UAS-DInR*-Δ*ABC*. Top row **(A,D,G,J)**, photographs of eyes with light microscopy. Note that expression of DInR results in increased eye volume such that the eyes now have increased depth. These eyes project out from the 2D image, occupying a larger 3D space. Middle row **(B,E,H,K)**, SEM. Bottom row **(C,F,I,L)**, high power SEM images show rough eyes induced by ectopic DInR expression. **(M,N)** Moderate expression using an *arm-GAL4* driver. **(M)** Third instar larvae: control, *UAS-DInR*, *UAS-DInR-KA*; **(N)** pupae: control, *UAS-DInR*, *UAS-DInR-KA*.

Based upon the findings summarized above, we generated a series of transgenes carrying mutations or deletions of sites critical for kinase activity or adapter protein binding in the context of the full-length *dinr* coding region (Figure 1B, Table 1). A C-terminal 3xMyc tag was added to each *dinr* variant for antibody detection and each *dinr* cDNA was inserted into a *pUAST* vector for expression with the *GAL4/UAS* system (Brand et al., 1994). After P-element mediated transformation, multiple independent transformant lines were obtained for each transgene and expression levels were quantitated. To verify the function of full-length DInR in tissue growth, and to test whether the 3xMyc tag interferes with DInR function, DInR-MYC3 was expressed in the developing eye using a moderate *ey-GAL4* driver. This resulted in cell autonomous overgrowth of the eye (Figures 3A–F). The increased eye volume resulted in eyes occupying larger volumes with increased depth, not always clearly depicted in two-dimensional images of whole eyes. This eye size difference appeared to be due to an increase in both cell size and cell number. In contrast, and as expected, a DInR transgene carrying a point mutation in the ATP binding site (“kinase-dead” mutation; DInR-K1405A) appeared to function as a dominant negative in these experiments, resulting in smaller eyes (Figures 3G–I). Similarly, expression of DInR-ΔABC resulted in smaller eyes (Figures 3J–L).

**Table 1:** **Rescue of adult lethality by DInR transgenes**.

**Transgene expressed**	**Rescued *dinr^ex15/273^* adults (% non-Sb)**	***dinr^ex15^*/+** and ***dinr^273^*/+ adults (% Sb)**	**Total no. of flies scored**
*UAS-lacZ*	0	100	237
*UAS-dinr*	37	63	785
*UAS-dinr-KA*	0	100	34
*UAS-dinr*-Δ*CD*	27	73	271
*UAS-dinr*-Δ*AB*	0	100	242
*UAS-dinr-Y1F*	0	100	245
*UAS-dinr-Y2F*	8	92	153
*UAS-dinr-LESL*	42	58	177
*UAS-dinr-LESL,Y2F*	11	89	254
*UAS-dinr-JM-NPFF*	24	76	213
*UAS-dinr-Y9F*	16	84	145
*UAS-dinr-Y7,8,9,10F*	11	89	235
*UAS-dinr-5NPXF*	21	79	246

Overexpression of full-length DInR in a wild type background with a moderate ubiquitous driver, *arm-GAL4*, caused whole animal overgrowth evident at larval and pupal stages (Figures 3M,N). Expression of DInR-KA acted as a dominant negative on whole animal growth.

To test the ability of DInR transgenes to complement wild type functions of DInR, transgenes were expressed in a *dinr^ex15/273^* mutant background under the control of an *arm-GAL4* driver. These *dinr^ex15/273^* transheterozygotes carry one copy of the *dinr^ex15^* null allele and one copy of the *dinr^273^* weak hypomorphic allele. To test for rescue, *arm-GAL4/arm-GAL4;FRT82Bdinr^273^/TM3Sb,armGFP* virgin females were crossed to *UAS-X/(UAS-X* or *CyO);dinr^ex15^/TM3Sb,armGFP* males and rescue of lethality was scored by counting non-Sb adults. As shown in Table 1, a negative control protein, β-galactosidase, encoded by the *UAS-lacZ* transgene, failed to rescue viability, as expected. However, viability to adulthood was fully rescued by wild type DInRMYC3 (*UAS-dinr*). *UAS-dinr-KA*, encoding a “kinase-dead” receptor, failed to rescue viability, consistent with the expectation that kinase activity is necessary for DInR function *in vivo*.

### Rescue of *dinr*-associated lethality by DInR mutant transgenes

To test the importance of the Dock and Chico binding sites identified *in vitro* (Figure 2 and (Poltilove et al., 2000)) for DInR function *in vivo*, the rescue approach described above was used. Mutations were introduced into full-length *pUASTDInRMYC3*, as described in the Materials and Methods. The mutant proteins were designed to test *in vivo* requirements for different DInR sequences: (1) DInR-KA, mutation of K1405 to A in the ATP binding site (“kinase-dead”). (2) DInR-Δ*CD*, deletion of the C and most of the D regions of the C-tail. (3) DInR-Δ*AB*, deletion of the A and B regions of the C-tail and part of the adjoining kinase domain, N-terminal of the C-tail. (4) DInR-Y1F, mutation of Y1714 in the A region of the C-tail. (5) DInR-Y2F, mutation of Y1776 in the B region of the C-tail. (6) DInR-LESL was designed to test the role of the potential SH3 binding PXXP motif in region A of the DInR C-tail. (7) DInR-LESL, Y2F, mutation of the PXXP in the A region and of Y1776 in the B region. (8) DInR-JM-NPFF, mutation to F of Y1354, embedded in an NPFY motif in the juxtamembrane region, previously shown to be required for Chico interaction (Poltilove et al., 2000). (9) DInR-Y9F, mutation of one of four Chico binding sites in the C region of the C-tail. (10) DInR-Y7,8,9,10F, simultaneous mutation of all four Chico binding sites in the C region of the C-tail. (11) DInR-5NPXF (JM-NPFF,Y7,8,9,10F), simultaneous mutation of the juxtamembrane NPFY and the four Chico binding sites in region C of the C-tail. *dinr* cDNAs encoding the DInR proteins described above were inserted into the P-element vector *pUAST*. Multiple independent transformant lines were generated for each and insertions on chromosome II were selected for rescue experiments. DInR proteins were expressed with the *GAL4/UAS* system, using a moderate, ubiquitous *arm-GAL4* driver.

As shown in Table 1, the CD region of the C-tail was not required for rescue to adulthood, as *UAS-dinr*-Δ*CD* expression rescued viability. In contrast, the AB region, containing the N-terminal half of the C-tail and a small portion of the conserved kinase domain, was required to support viability, as no adults were observed when *UAS-dinr*-Δ*AB* was expressed in the *dinr^ex15/273^* mutant background. Interestingly, DInR-Y1F completely failed to rescue adult lethality of *dinr^ex15/273^* transheterozygotes, indicating that tyrosine 1714, within the A region of the C-tail, is crucial for adult viability. Expression of *UAS-dinr-Y2F* rescued a small number of animals, suggesting that this residue contributes to but is not absolutely required for viability. The PESP motif in the A region of the C-tail did not appear to be required for viability, as indicated by rescue with *UAS-dinr-LESL* and with *UAS-dinr-LESL,Y2F*, the latter showing similar rescue potential to the Y2 mutation alone. Mutation of the juxtamembrane NPFY tyrosine alone had little effect on viability as DInR-JM-NPFF rescued lethality substantially. Mutations in individual or multiple candidate Chico binding sites, DInR-Y9F and DInR-Y7,8,9,10F rescued lethality to a lesser degree. Finally, DInR-5NPXF, a compound variant containing mutations of the juxtamembrane NPFY tyrosine and all candidate Chico binding sites in the C-tail rescued viability. In sum, most DInR proteins carrying small deletions and specific mutations retained the ability to complement loss of DInR function, rescuing mutants to adulthood. The exceptions to this were DInR-ΔAB and DInR-Y1F. Since the latter targets only one tyrosine residue in the A region of the C-tail and was required for viability, this probably accounts for the failure of DInR-ΔAB to rescue lethality.

### Chico interaction sites are required for DInR's growth function

**Figure 4:**
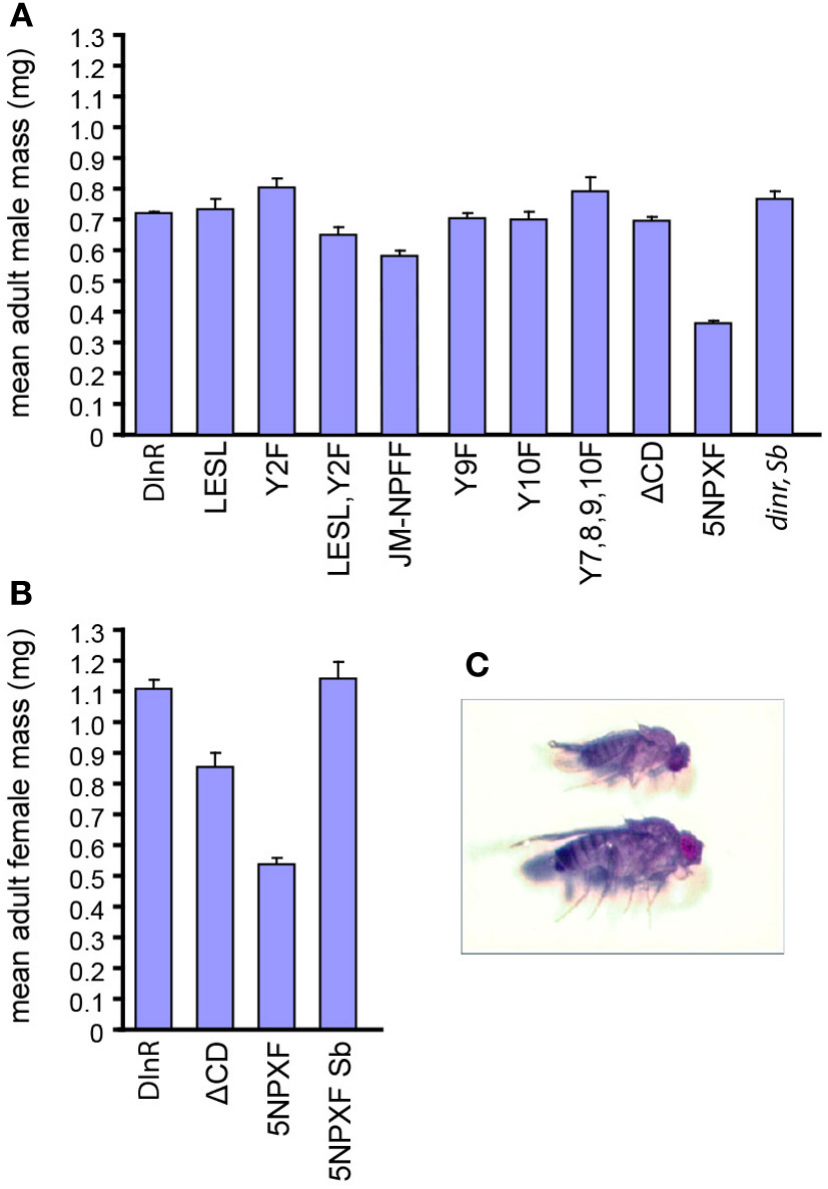
**Chico interaction sites are required for whole animal growth. (A,B)** Mean mass (mg) of male **(A)** and female **(B)** adult *dinr^ex15/273^* flies expressing different UAS-DInR variants. Error bars represent standard error. Sample numbers for each rescue protein tested in males were: DInR control, 85; DInR-LESL, 10; DInR-Y2F, 5; DInR-LESL,Y2F, 4; DInR-JM-NPFF, 33; DInR-Y9F, 10; DInR-Y10F, 5; DInR-Y7,8,9,10F, 2; DInR-ΔCD, 27; DInR-5NPXF, 24; DInR control expressed in *dinr* mutant heterozygote siblings, 4. Sample numbers for each rescue protein tested in females were: DInR control, 32; DInR-ΔCD, 8; DInR-5NPXF, 9; DInR-5NPXF expressed in *dinr* mutant heterozygote siblings, 3. **(C)** Photograph showing an example of a small *dinr* mutant adult fly expressing DInR-5NPXF (top) compared to heterozygous sibling control expressing DInR-5NPXF (bottom).

We next investigated the size of animals rescued by different DInR variants, with the expectation that animals carrying mutations in Dock binding sites would be similar in size to animals rescued by full-length DInR, while those lacking Chico interaction sites would be similar to small *chico* mutants. As shown in Figure 4A, the size of male transheterozygotes expressing full-length DInR was similar to that of sibling heterozygote controls expressing full-length DInR (*dinr, Sb*). Mutations in the putative Dock binding sites did not have detrimental effects on growth; DInR-LESL and DInR-Y2F rescued adult male growth, although DInR-LESL,Y2F animals were somewhat smaller. Mutation of the juxtamembrane NPFY tyrosine (DInR-JM-NPFF) led to an incomplete rescue of growth (note that an average of two independent lines is shown for this transgene; for all other transgenes, results are shown for a single transformant line). Mutation of one or more Chico binding sites in the C-tail did not have detrimental effects on growth: DInR-Y9F, DInR-Y10F and DInR-Y7,8,9,10F fully rescued growth defects, as did DInR-ΔCD, which lacks the C-terminal half of the C-tail containing Y7, 8, 9, and 10. However, there was a 50% decrease in the mean mass of adult males rescued by DInR-5NPXF compared to those rescued by the DInR control. This decrease is very similar to the 55% decrease in body weight seen in *chico* mutant males (Bohni et al., 1999). Thus, it seems likely that these five NPXY sites are responsible for most, if not all, of the control of growth by DInR that is mediated by Chico, and that there is considerable redundancy among these NPXY sites.

In females, there was a 52% decrease in the mean mass of adult females rescued by DInR-5NPXF compared to those rescued by the control DInR protein (Figures 4B,C). This was noticeably less than the 65% decrease in body weight measured in *chico* mutant females (Bohni et al., 1999). Interestingly, although the DInR-ΔCD protein, which lacks the four NPXY sites in the C-tail in addition to two other tyrosines and one PXXP site, rescued the mean mass of adult males to the same level as the control DInR protein, this did not occur for adult females; instead, there was a 23% decrease in mean mass in females rescued by the DInR-ΔCD protein. Thus, it is possible that another interaction site(s) in regions C and D, other than the four NPXY sites, may also be responsible for growth control through Chico in females. Since DInR is also involved in controlling female fertility [reviewed in Garofalo (2002)], an interplay between fertility and growth may be at work.

### Mutation of chico interaction sites did not abolish rescue of *dinr*-associated axon guidance defects

**Figure 5:**
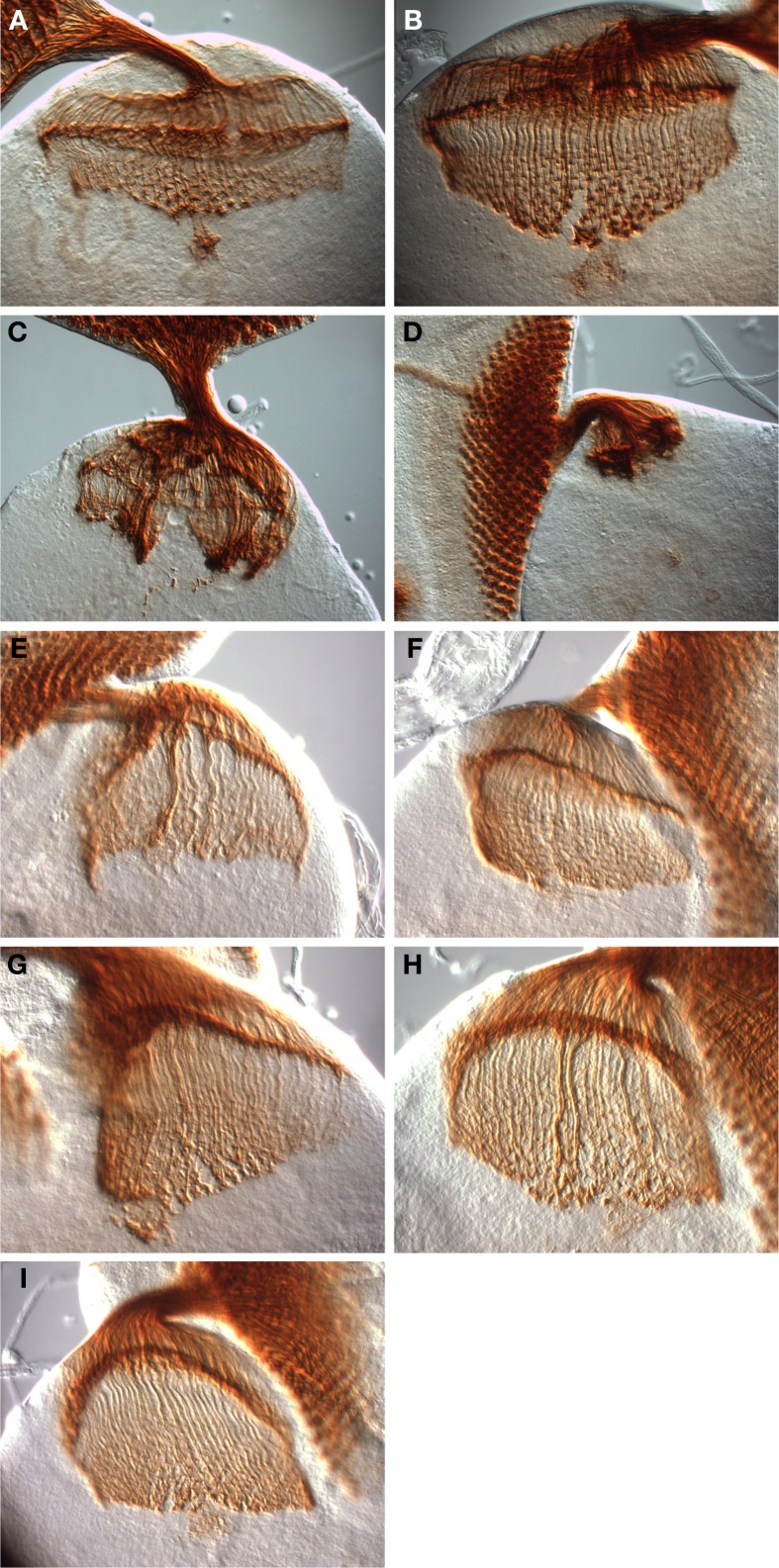
**Rescue of *dinr*-associated axon guidance defects by full-length DInR and variants.** Third instar larval eye-brain complexes were stained with MAb24B10 to visualize differentiated photoreceptors and their axons. Imaging was done with DIC optics and a 40× objective. **(A,B)** Control eye-brain complexes of the genotype *w^1118^*. The specimen shown in **(B)** is at a later stage of development than that shown in **(A)**. The rip in the medulla in **(B)** resulted during sample preparation. **(C,D)** Axon guidance was severely disrupted in eye-brain complexes from whole animal *dinr^ex15/273^* transheterozygotes. **(E)** Axon guidance defects were rescued with full-length DInR. **(F–I)** Axon guidance defects were rescued by expression of DInR variants: **(F)** DInR-LESL; **(G)** DInR-Y2F; **(H)** DInR-LESL, Y2F; **(I)** DInR-5NPXF.

As shown in Figure 5, severe axon guidance defects were observed in *dinr^ex15/273^* transheterozygotes, which display gross disorganization of photoreceptor axon targeting in both the lamina and medulla (compare wild type eye-brain complexes, Figures 5A,B, to *dinr^ex15/273^* eye-brain complexes, Figures 5C,D). Clumps of axons were present above and in the lamina, and in the medulla. In general, the phenotypes observed in the *dinr^ex15/273^* eye-brain complexes were more severe than those of *dinr^353/273^* (Song et al., 2003). This is consistent with *dinr^ex15^*, a null allele (Song et al., 2003), being a stronger loss-of-function allele than *dinr^353^*. Importantly, these axon guidance defects were largely rescued by expression of full-length DInR (Figure 5E). This is the first demonstration that axon guidance defects are rescued by DInR, ruling out the possibility that defects seen in *dinr* mutants result from the genetic background of the *dinr* mutants. Surprisingly, the DInR variants carrying mutations in candidate Dock binding sites also rescued axon guidance defects. These include DInR-LESL (Figure 5F), in which one of the putative Dock binding sites was mutated, DInR-Y2F (Figure 5G), in which the second putative Dock binding site was mutated, and DInR-LESL, Y2F (Figure 5H), in which both putative Dock binding sites were mutated. Notably, DInR-5NPXF, which did not rescue growth defects (see above, Figure 4), did restore normal photoreceptor axon guidance (Figure 5I). This rescue by DInR-5NPXF demonstrates that Chico interaction sites are not required for axon guidance functions of DInR. Furthermore, this shows that *dinr*-associated axon guidance defects are not merely a secondary effect of *dinr*-associated growth defects.

## Conclusions

Like other RTKs, DInR regulates multiple processes, including growth and axon guidance. Here we have tested the hypothesis that DInR interacts differentially *in vivo* with different adapter proteins to mediate different biological functions. In cell-based assays, different regions of DInR interact with Chico and Dock, adapter proteins implicated in growth and axon guidance, respectively. Using *in vivo* rescue experiments, we found that mutations in DInR's Chico interaction sites rescued viability and axon guidance defects, but, as expected, animals were small, similar to *chico* mutants. This finding supports the notion that DInR interacts directly with Chico to control growth during *Drosophila* development. In contrast, expression of both wild type and mutant DInR proteins rescued axon guidance defects of *dinr* mutants. Rescue by wild type DInR confirmed its role in photoreceptor axon targeting. However, it was not expected that DInR variants with mutations in Dock binding sites (Y2, LESL) would rescue axon guidance. At first glance, these results appear to suggest that Dock interaction with DInR is not required for DInR function in axon guidance. However, several other explanations are possible. For example, the single tyrosine identified in our studies that is required for viability (Y1) may also be required for DInR's role in axon guidance. Alternatively, Dock may be capable of binding to multiple sites in DInR *in vivo*, possibly including the many candidate tyrosines and PXXPs in the C-tail (Figure 1B). This would lead to functional redundancy *in vivo*, providing a high degree of buffering to ensure interaction between the two proteins and proper axon targeting. In sum, our studies demonstrate that DInR utilizes different protein domains, and likely different adapter proteins, to segregate signaling in axon guidance and growth.

## Author contributions

Caroline R. Li and Dongyu Guo designed and carried out experiments and analyzed data. Leslie Pick designed experiments and oversaw experiments. Leslie Pick and Caroline R. Li wrote the paper.

### Conflict of interest statement

The authors declare that the research was conducted in the absence of any commercial or financial relationships that could be construed as a potential conflict of interest.
